# Supplementary material for: Rapid turnover of life-cycle-related genes in the brown algae
Source: Genome Biol. 2019 Feb 14;20:35. doi: 10.1186/s13059-019-1630-6 (PMC6374913; doi:10.1186/s13059-019-1630-6)
Supplement: Supplementary file 3 — Protein alignments, corresponding Gblocks html file and CDS sequences for the lineage conserved gametophyte-biased genes. (ZIP 2109 kb) [file 13059_2019_1630_MOESM3_ESM.zip › Con_GA_alignments/Ec-19_004770.aa_ali.fasta-gb.htm]

Ec-19\_004770.aa.fasta.trans.aa\_ali.fasta


## Gblocks 0.91b Results

Processed file: **Ec-19\_004770.aa.fasta.trans.aa\_ali.fasta**  
Number of sequences: **2**  
Alignment assumed to be: **Protein**  
New number of positions: **182** (selected positions are underlined in blue)

```
                         10        20        30        40        50        60
                 =========+=========+=========+=========+=========+=========+
Ec-19_004770     MKRPDPTGNDDDDDEEEEEVRTLVVRAIDREWVMGEDLILPVTGDETIVEIQQVIKCMKG
MSTRG.36901.1    -QQGSPKG-----DEEGEDTRTLVVRAIDGEWIMGDDLILTVTGDETIVEIQQVIKSMKG
                              ###############################################


                         70        80        90       100       110       120
                 =========+=========+=========+=========+=========+=========+
Ec-19_004770     VPLTRMVAFKEESESRISDKHMTWSLSRNGLYAGAVLVLRPSRPNQWLWHPLEWYEERLL
MSTRG.36901.1    VPITRMVAFKEEQEARISDKHMTWSLSRNGLYDGEVLVIRPSRPNQWLWHPLEWYEERLL
                 ############################################################


                        130       140       150       160       170       180
                 =========+=========+=========+=========+=========+=========+
Ec-19_004770     KAAEDVASSNPWAEMGTGCPLSILEKEVLVPPPLRRDSLKGILRRFPDRLRLQLDVVSQK
MSTRG.36901.1    KEAEAVASSSPWVEWGTGCPLSALQKKVLVPPPLSRDSLKGILRKFPDRLRLQLDVVSQE
                 ############################################################


                        190
                 =========+======
Ec-19_004770     FVAFPNKDEMLLPSSY
MSTRG.36901.1    FVAFPNKDNLLLPSSF
                 ###############
```

```
Parameters used
Minimum Number Of Sequences For A Conserved Position: 2
Minimum Number Of Sequences For A Flanking Position: 2
Maximum Number Of Contiguous Nonconserved Positions: 8
Minimum Length Of A Block: 10
Allowed Gap Positions: None
Use Similarity Matrices: Yes
```

```
Flank positions of the 1 selected block(s)
Flanks: [14  195]  

New number of positions in Ec-19_004770.aa.fasta.trans.aa_ali.fasta-gb:  182  (92% of the original 196 positions)
```
